# Supplementary material for: RAPDOR: Using Jensen-Shannon Distance for the computational analysis of complex proteomics datasets
Source: Nat Commun. 2025 Sep 26;16:8527. doi: 10.1038/s41467-025-64086-7 (PMC12475003; doi:10.1038/s41467-025-64086-7)
Supplement: Supplementary file 1 — Supplementary Information [file 41467_2025_64086_MOESM1_ESM.pdf]

Supplementary Figures

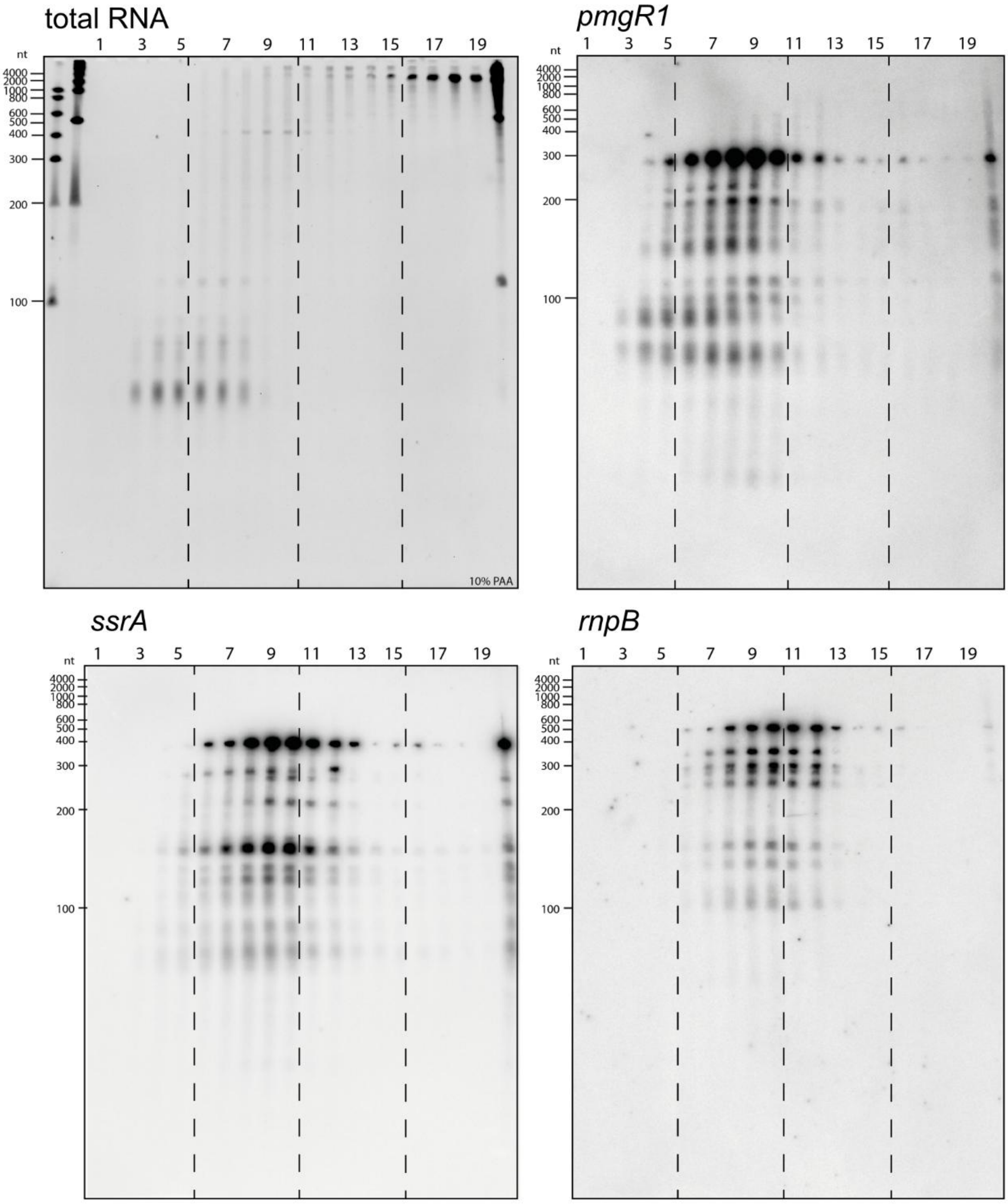

Supplementary Figure 1 Distribution of known RNA species in the untreated control samples. Northern blots of 10% denaturing PAA gels hybridized with radioactively labelled probes. Gel image of total RNA prepared from the different gradient fractions as numbered on top. The molecular mass standards in the left two lanes were the High and Low Range RiboRuler RNA Ladders (Thermo Fisher Scientific). Hybridization is shown against the sRNA PmgR1[1], the transfer-messenger RNA (tmRNA) SsrA[2] and RNase P RNA RnpB[3]. Source data are provided as a Source Data file.

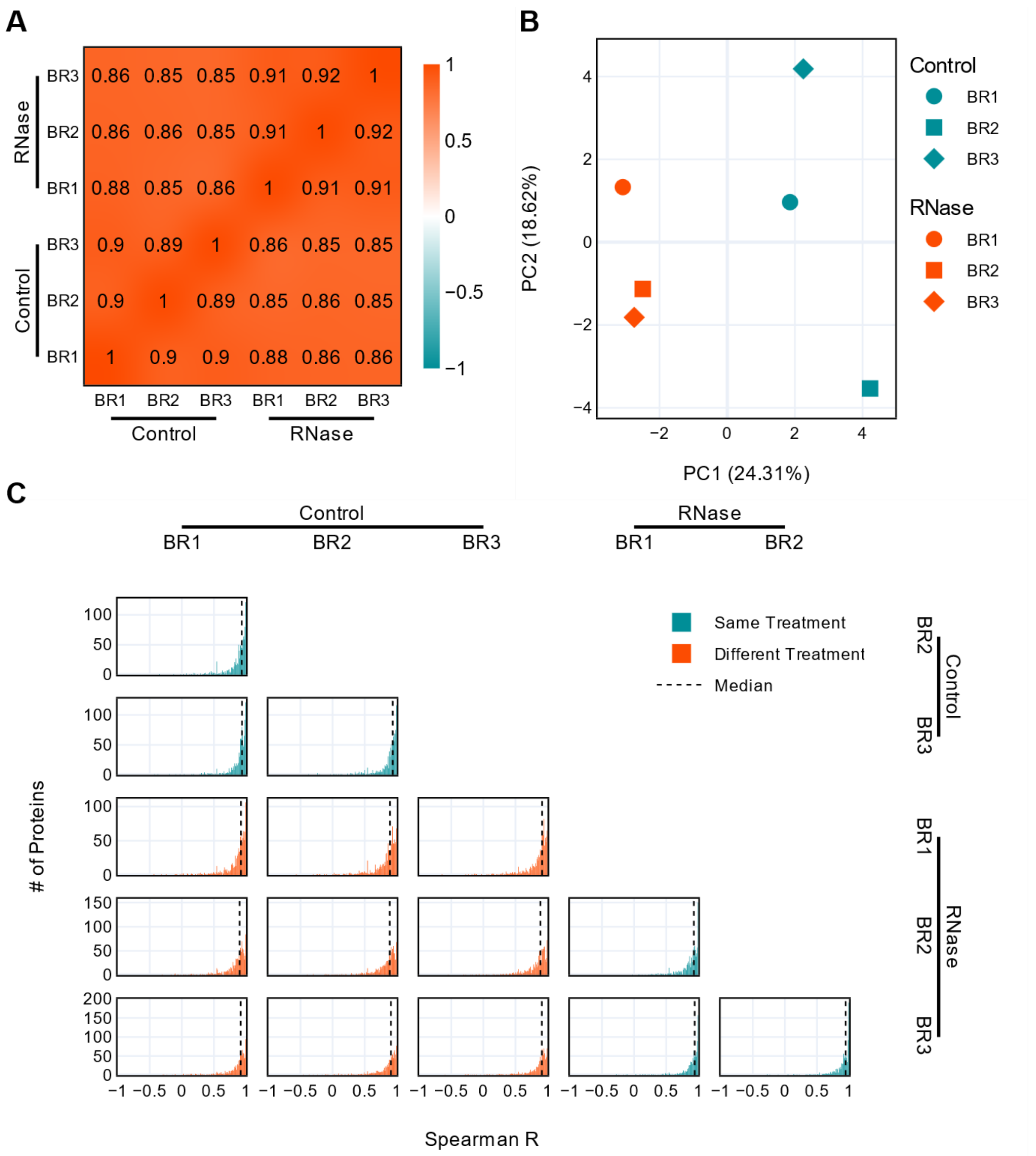

Supplementary Figure 2 Quality control of the *Synechocystis* 6803 GradR dataset. (A) Spearman correlation of IBAQ values between each sample combination. **(B)** PCA of iBAQ values. Each protein-fraction combination was treated as a separate feature. The top 20% of protein-fraction entries with the highest variance across all samples were selected for dimensionality reduction. **(C)** Histograms of Spearman correlation of sample-wise individual protein distribution profiles.

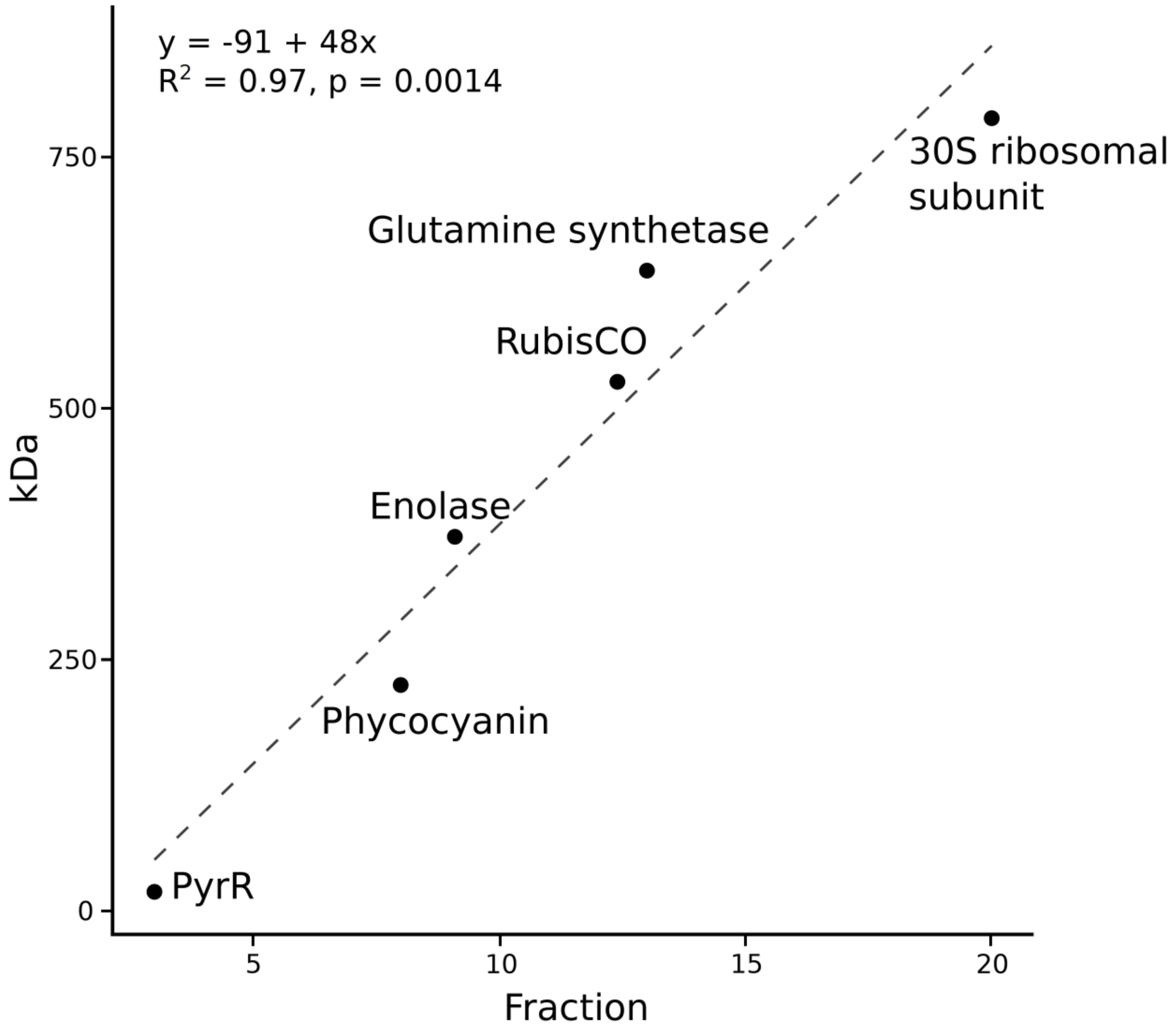

Supplementary Figure 3 Resolution of gradients. Sedimentation of selected proteins and protein complexes in specific fractions of a sucrose density gradient after ultracentrifugation (x-axis) in comparison to the calculated molecular mass (y-axis). Individual protein complexes often span several adjacent fractions. Therefore, the respective peak fractions of the indicated protein complexes were selected for the calibration curve. Masses were calculated for an α3β3 hexameric phycocyanin complex[4], for RubisCO consisting of an 8 small and 8 large subunits[5], for the homo 8-mer enolase *of Synechococcus elongatus*[6], the homo 12-mer glutamine synthetase[7] and 30S ribosomal subunit of *E. coli*[8]. PyrR (Sll0368) is the bifunctional *pyr* operon transcriptional regulator/uracil phosphoribosyltransferase PyrR (molecular mass 19.946 kDa).

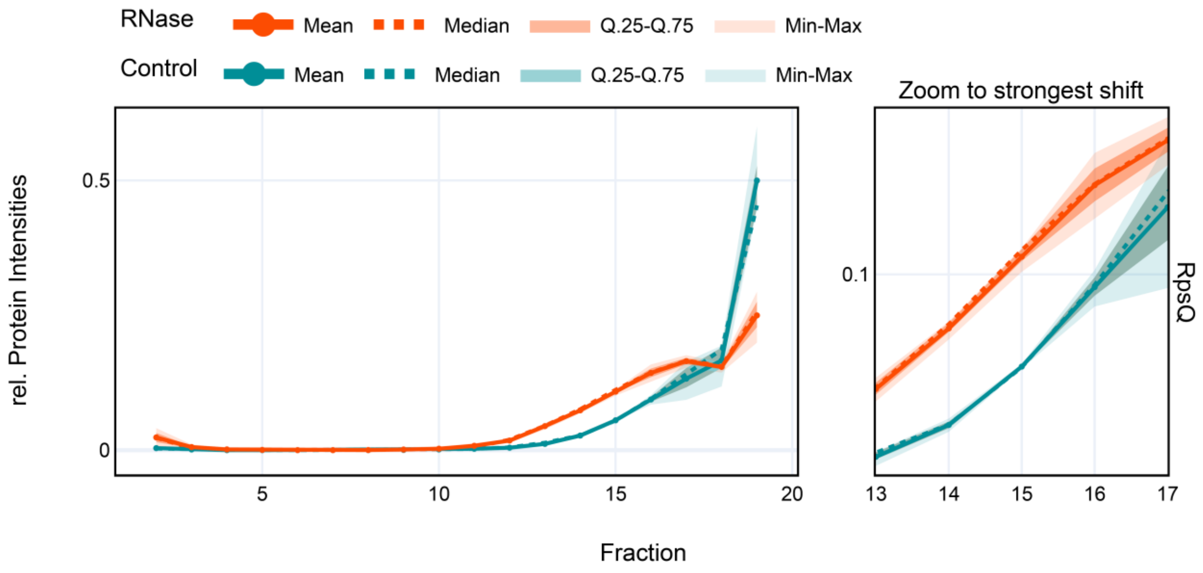

Supplementary Figure 4 Redistribution of RpsQ. Like most small ribosomal subunit proteins, RpsQ showed a redistribution from the last fraction to nearby lower molecular mass fractions. This resulted in a broader peak that was identified as a significant shift by RAPDOR, but not by the R-DeeP analysis pipeline. Source data are provided as a Source Data file.

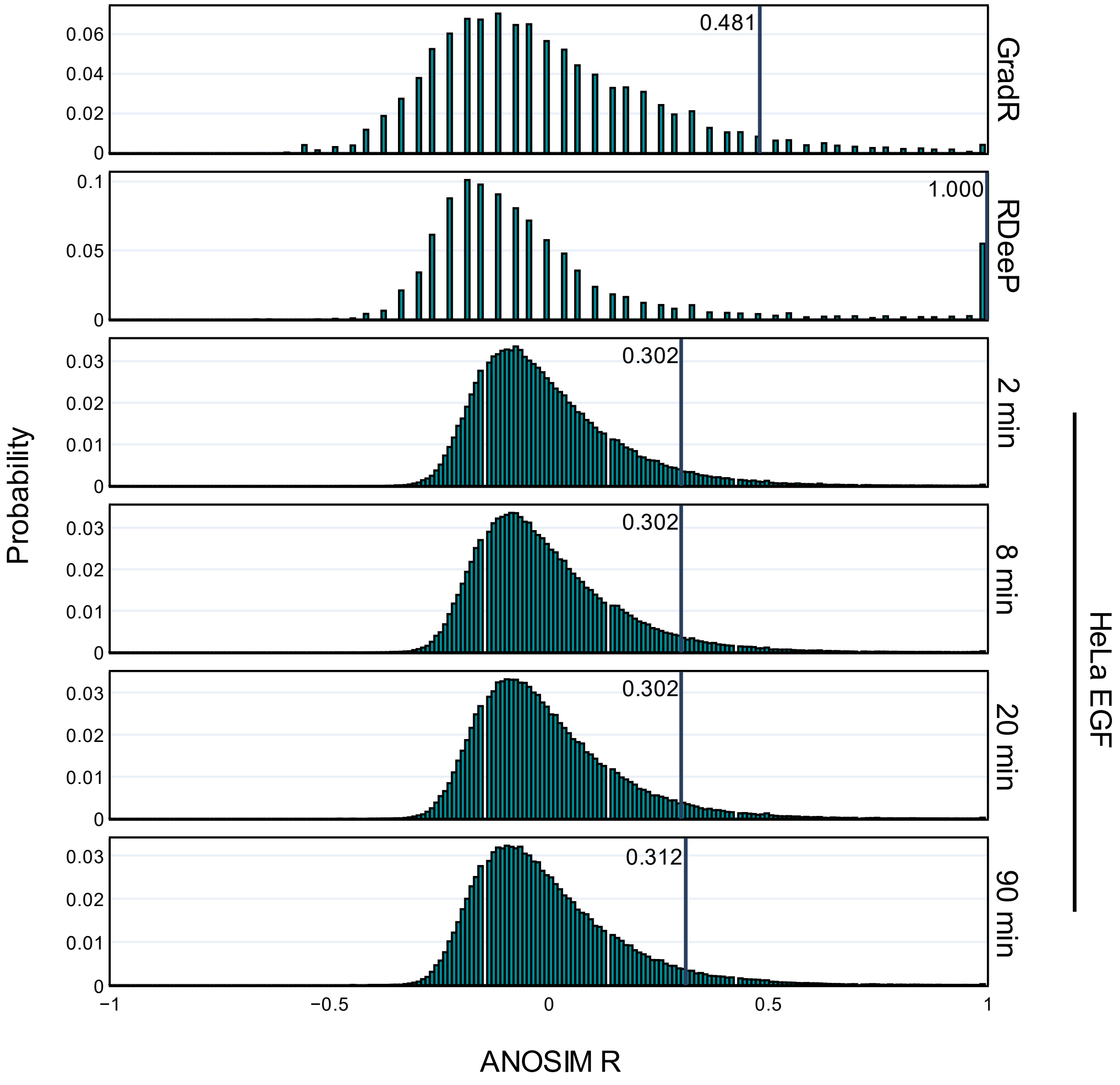

Supplementary Figure 5 Distribution of ANOSIM *R* values of the different datasets. Distributions were generated using all possible permutation of treatment labels and calculating the ANOSIM *R* value. Afterwards, the values of different proteins with signal in all replicates were stacked together to generate the histograms of the background distributions. This method assumes that *R* values for different proteins follow a similar distribution. Vertical lines indicate the 95 percentile. The first dataset is from this study. The second row contains the dataset from the original R-DeeP publication[9]. The EGF treated HeLa datasets correspond to different treatment time points from Martinez-Val et al.[10]. Source data are provided as a Source Data file.

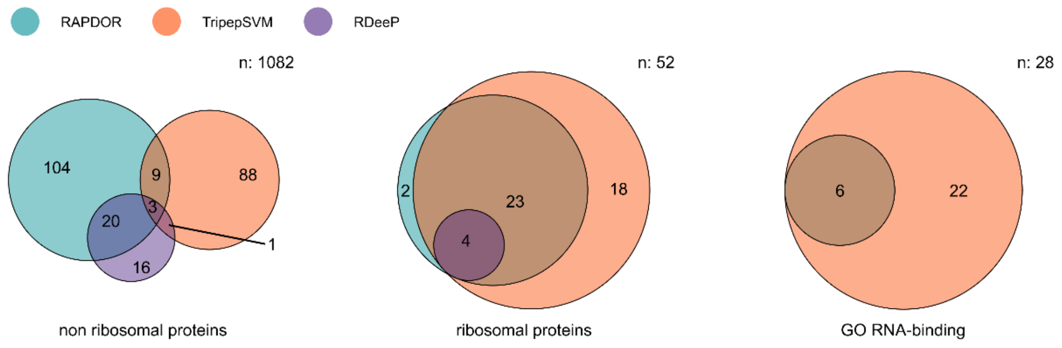

Supplementary Figure 6 RNA-dependent proteins identified via the different approaches. The respective total numbers of proteins identified by mass spectrometry are indicated as n values. The first Venn diagram shows the numbers of RNA-dependent proteins excluding ribosomal proteins identified by the different approaches. The number of ribosomal proteins identified as RNA-dependent via the different approaches is displayed in the middle. The right panel summarizes all identified proteins with the RNA-binding GO annotation that were not ribosomal proteins.

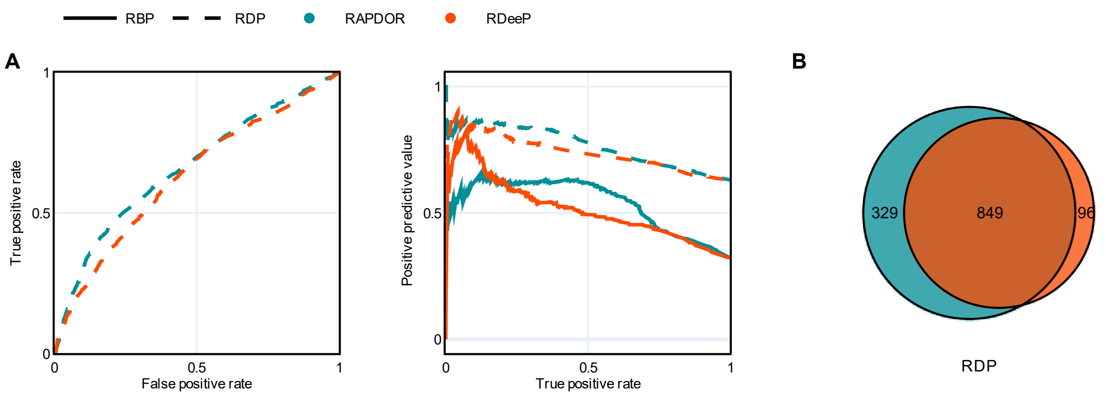

C

Tool	RBP-AUROC	RBP-AUPRC	RDP-AUROC	RDP-AUPRC
RAPDOR	0.73	0.54	0.66	0.76
RDeeP	0.69	0.52	0.63	0.74

Supplementary Figure 7 Comparison of RAPDOR and R-DeeP regarding RDP classification. (A) AUROC for RDP classification and AUPRC for RBP and RDP classification of both tools. **(B)** Number of proteins identified by the two approaches that are either RBPs themselves or have a high confidence protein-protein interaction >0.7 in the STRING database[11]. **(C)** Performance measures (numerical values) of RBP and RDP classification for the RAPDOR and R-DeeP tool. Source data are provided as a Source Data file.

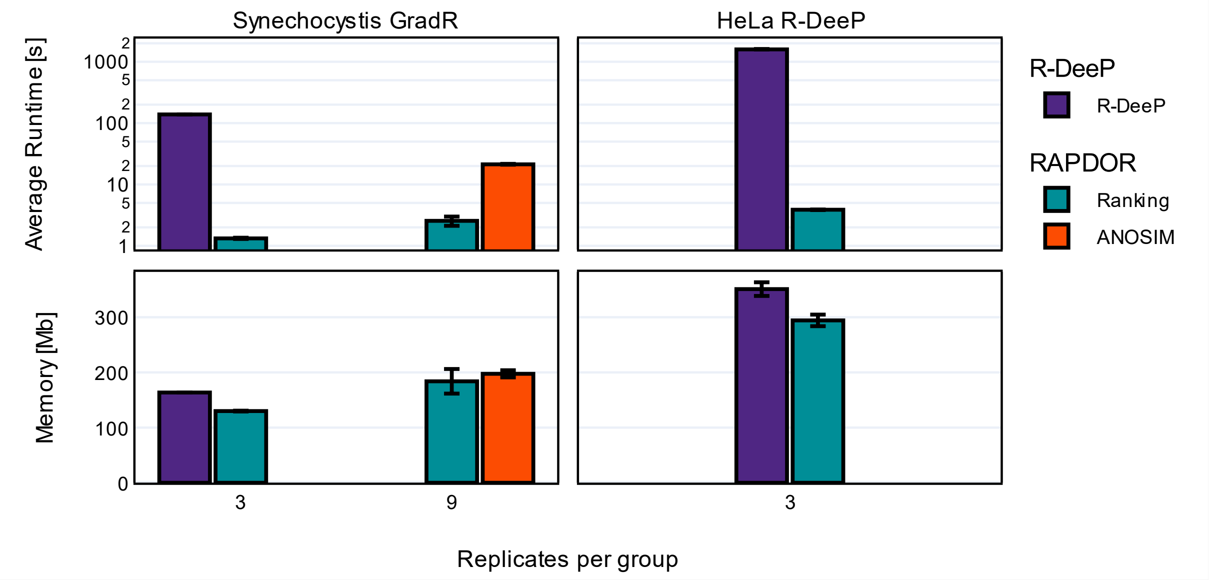

Supplementary Figure 8 Runtime comparison of the tools. Average runtime and memory consumption of 5 runs of each tool for the indicated number of replicates per group. The left column contains the comparison for the *Synechocystis* 6803 GradR data and the right column displays values for the HeLa R-DeeP dataset. Note that the y-axis on runtime is on log scale. Source data are provided as a Source Data file.

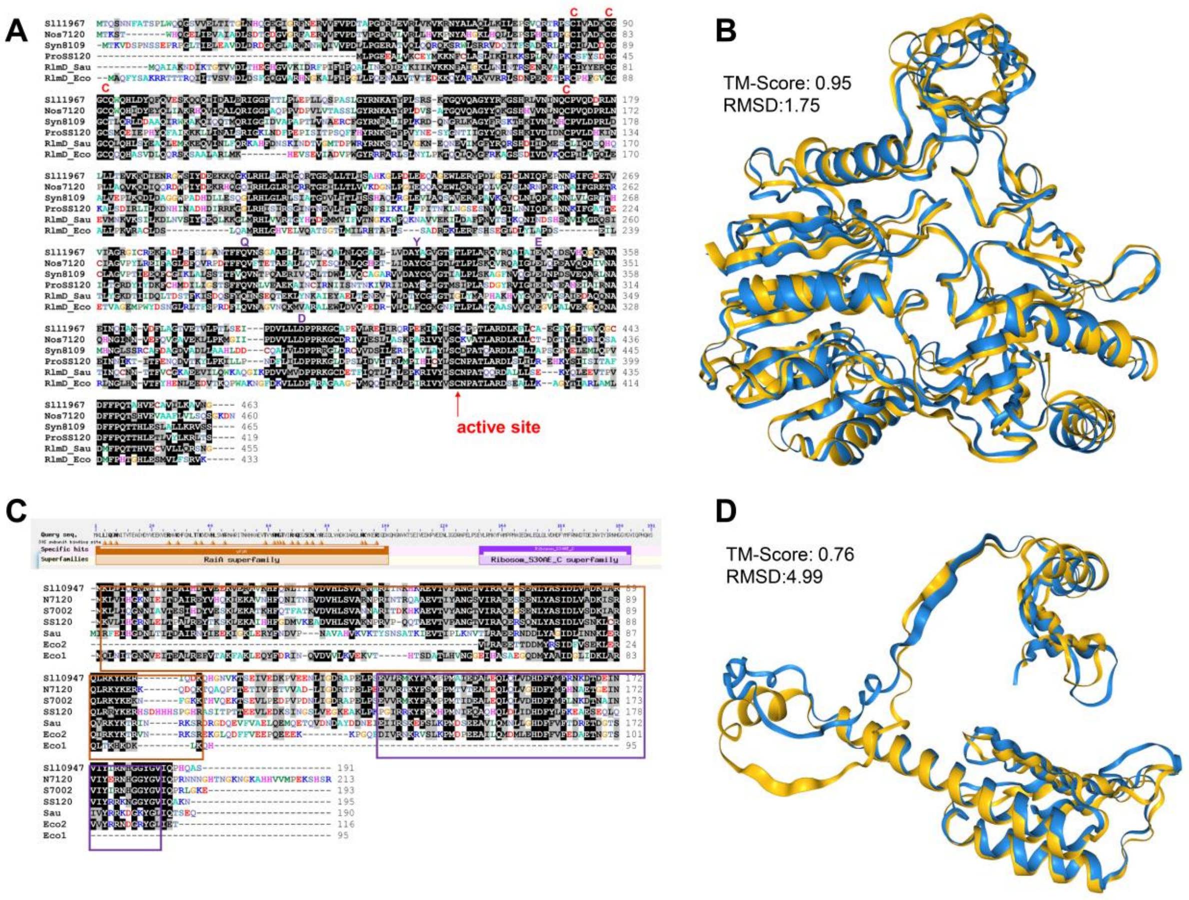

Supplementary Figure 9 Comparison of RBPs from *Synechocystis* 6803 to homologs from other bacteria. A. Putative rRNA uracil(1939)-C(5))-methyltransferase Sll1967 multiple sequence alignment with homologs from the cyanobacteria *Nostoc* 7120 (WP_010997798), *Synechococcus* sp. WH 8109 (WP_006851074), *Prochlorococcus marinus* CCMP1375 (AAQ00010), and from *Staphylococcus aureus* (WP_031912837), and *E. coli* (WP_001521172). **B.** Structural comparison of Sll1967 with the *Staphylococcus aureus* subsp. aureus NCTC 8325 RNA methyltransferase RlmD. **C.** Multiple sequence alignment of the putative ribosome-associated translation inhibitor RaiA/LrtA Sll0947 with homologs from the cyanobacteria *Nostoc* 7120 (WP_010997335), *Synechococcus* sp. PCC 7002 (WP_012305897), *Prochlorococcus marinus* CCMP1375 (WP_011124552), and from *Staphylococcus aureus* (WP_000617735), *E. coli* 2 (WP_137516021) and *E. coli* 1 (WP_275231370). Sll0947 consists of two different domains, a RaiA superfamily domain (boxed in brown) and a Sigma 54 modulation/S30EA ribosomal protein C terminal domain (boxed in purple). These domains are found in two separate proteins in *E. coli*. **D.** Structural comparison of Sll0947 (blue) with the *Staphylococcus aureus*

subsp. aureus NCTC 8325 ribosome hibernation promotion factor (yellow). The respective TM scores and RMSD values given in panels C and D support the structural similarity between the compared protein structures[12].

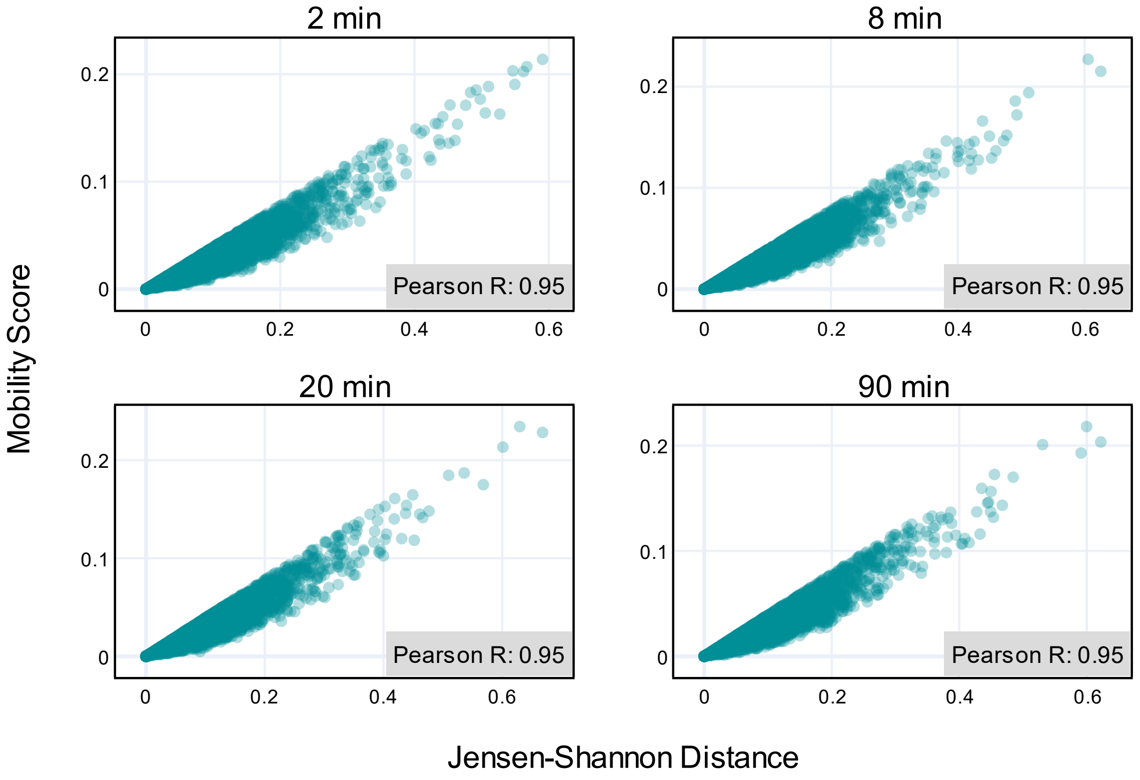

Supplementary Figure 10 Comparison of Jensen-Shannon distance and the mobility score calculated by Martinez-Val, et. al.[10]. The Jensen-Shannon distance and the mobility score showed a strong Pearson correlation for all four comparisons. Source data are provided as a Source Data file.

Supplementary Table

Supplementary Table 1. Deoxynucleotide primers and plasmids used in this work. All primers were purchased from IDT. The names of sRNAs are given as previously defined[13]. All sequences are in 5' to 3' direction. Added T7 promoter sequences are indicated by lowercase letters.

**Oligonucleotide primers**		
**Primer**	**Name or purpose**	**Sequence**
ncr0700_probefd	Amplification of probe templates to detect sRNA PmgR1 by Northern hybridization.	CTTCACTGGCAGATAAAAAC
ncr0700_probe_T7rv		taatacgactcactatagggGATGAACTGAGAAACAAACG
ncl1680_probe_fd	Amplification of probe templates to detect tmRNA/SsrA by Northern hybridization.	AATGGTTTCGACAGGTTGGC
ncl1680_probe_T7rv		taatacgactcactatagggCCCGTTTGAAGCTGACGATG
rnpB_probe_fw	Amplification of probe templates to detect RNase P RNA/RnpB by Northern hybridization.	AGTTGCGGATTCCTGTCACAG
rnpB_probe_T7rv		taatacgactcactatagggGTGGCACTGTCCTCACGCTC
P1	pUC19_fwd_shorterback bone2	AGCTCACTCAAAGGCGGTAA
P2	pUC19_rev_shorterback bone2	TCACCGTCATCACCGAAACG
P3	pUC19_pPetE_fwd	CGTTTCGGTGATGACGGTGAGAAGGGATAGCAAGCTAATTTTTATG
P4	oop-**pUC19s_**Rev	**TTACCGCCTTTGAGTGAGCT**AATAAAAAACGCCCGGCG
P5	P*petE* rev	ACTTCTTGGCGATTGTATCTATAGG
P6	3 x FLAG_3'UTR_T*oop* fwd	GATTATAAAGATCATGATGG
P7	1_fwd	AGATACAATCGCCAAGAAGTATGACCCAAAGTAATAATTTCGC
P8	1_rev	CCATCATGATCTTTATAATCACCATTGACCGCTTTGAG
P9	2_fwd	AGATACAATCGCCAAGAAGTATGGGCACTAGTCCCCAAAATTC
P10	2_rev	CCATCATGATCTTTATAATCGGAAATTAGATCTTCTAAATCCCTGAGATTG
P11	3_fwd	AGATACAATCGCCAAGAAGTATGTCTATCAATGCTTACAAACTAG
P12	3_rev	CCATCATGATCTTTATAATCTAGGTGTCGGTATGCAGAATTATC
P13	4_fwd	AGATACAATCGCCAAGAAGTATGACCGAAACTACCATTAC
P14	4_rev	CCATCATGATCTTTATAATCACTAGCTTTTTCCTGATG
P15	5_fwd	AGATACAATCGCCAAGAAGTATGAAGCATATATTTAGGTTTAAAAATTATTTTTTCCATTC
P16	5_rev	CCATCATGATCTTTATAATCATTGGAGCCGGCCAATTTTC
P17	7_fwd	AGATACAATCGCCAAGAAGTATGTTTAAGCATTGCCTC
P18	7_rev	CCATCATGATCTTTATAATCACCTTTGGGAGAGAAAAAC
P19	9_fwd	AGATACAATCGCCAAGAAGTATGAAACTGTTAATTCAGGGCAATAATATCACAG
P20	9_rev	CCATCATGATCTTTATAATCGCTGGCTTGGTGGGGTTG
P21	10_fwd	AGATACAATCGCCAAGAAGTATGACAAGCAGAATTAATCCCC
P22	10_rev	CCATCATGATCTTTATAATCGCCCAAAGCCGAGGTAAC
P23	RP_PpetE_fwd	GCCGCCCGCATTGGAGAAATGAAGGGATAGCAAGCTAATTTTTATG
P24	RP_3xFLAG-oop_rev	GTGCAACGGGAATTTGAAGAAATAAAAAACGCCCGGCG
**Plasmids**		
**Name**	**Reference**	**Description**
X-54	This study	pUC19s:: P*petE-sll7087*-3xFLAG-*oop*
V-37	Not published	pVZ322:: P*petE-sll7087*-3xFLAG-*oop*
X-62	This study	pVZ322s:: P*petE-sll7067*-3xFLAG-*oop*
X-71	This study	pVZ322s:: P*petE-sll1371*-3xFLAG-*oop*
X-63	This study	pVZ322s:: P*petE-ssl2245*-3xFLAG-*oop*
X-64	This study	pVZ322s:: P*petE-slr0711*-3xFLAG-*oop*
X-65	This study	pVZ322s:: P*petE-sll1315*-3xFLAG-*oop*
X-66	This study	pVZ322s:: P*petE-slr0670*-3xFLAG-*oop*
X-67	This study	pVZ322s:: P*petE-sll0947*-3xFLAG-*oop*
X-68	This study	pVZ322s:: P*petE-sll0726*-3xFLAG-*oop*

Supplementary References

1. de Porcellinis, A. J. *et al.* The non-coding RNA Ncr0700/PmgR1 is required for photomixotrophic growth and the regulation of glycogen accumulation in the cyanobacterium *Synechocystis* sp. PCC 6803. *Plant Cell Physiol* **57**, 2091–2103 (2016).
2. de la Cruz, J. & Vioque, A. Increased sensitivity to protein synthesis inhibitors in cells lacking tmRNA. *RNA* **7**, 1708–1716 (2001).
3. Vioque, A. Analysis of the gene encoding the RNA subunit of ribonuclease P from cyanobacteria. *Nucleic Acids Res* **20**, 6331–6337 (1992).
4. Marx, A. & Adir, N. Allophycocyanin and phycocyanin crystal structures reveal facets of phycobilisome assembly. *Biochim Biophys Acta BBA - Bioenerg* **1827**, 311–318 (2013).
5. Cot, S. S.-W., So, A. K.-C. & Espie, G. S. A multiprotein bicarbonate dehydration complex essential to carboxysome function in cyanobacteria. *J Bacteriol* **190**, 936–945 (2008).
6. González, J. M., Martí-Arbona, R., Chen, J. C.-H. & Unkefer, C. J. The structure of *Synechococcus elongatus* enolase reveals key aspects of phosphoenolpyruvate binding. *Acta Crystallogr Sect F Struct Biol Commun* **78**, 177–184 (2022).
7. Mérida, A., Leurentop, L., Candau, P. & Florencio, F. J. Purification and properties of glutamine synthetases from the cyanobacteria *Synechocystis* sp. strain PCC 6803 and *Calothrix* sp. strain PCC 7601. *J Bacteriol* **172**, 4732–4735 (1990).
8. Maksimova, E. M. *et al.* RbfA is involved in two important stages of 30S subunit assembly: formation of the central pseudoknot and docking of helix 44 to the decoding center. *Int J Mol Sci* **22**, 6140 (2021).
9. Caudron-Herger, M. *et al.* R-DeeP: Proteome-wide and quantitative identification of RNA-dependent proteins by density gradient ultracentrifugation. *Mol Cell* **75**, 184-199.e10 (2019).
10. Martinez-Val, A. *et al.* Spatial-proteomics reveals phospho-signaling dynamics at subcellular resolution. *Nat Commun* **12**, 7113 (2021).
11. von Mering, C. *et al.* STRING: known and predicted protein–protein associations, integrated and transferred across organisms. *Nucleic Acids Res* **33**, D433–D437 (2005).
12. Xu, J. & Zhang, Y. How significant is a protein structure similarity with TM-score = 0.5? *Bioinformatics* **26**, 889–895 (2010).

13. Kopf, M. *et al.* Comparative analysis of the primary transcriptome of *Synechocystis* sp. PCC 6803. *DNA Res* **21**, 527–539 (2014).
