# Supplementary material for: RAPDOR: Using Jensen-Shannon Distance for the computational analysis of complex proteomics datasets
Source: Nat Commun. 2025 Sep 26;16:8527. doi: 10.1038/s41467-025-64086-7 (PMC12475003; doi:10.1038/s41467-025-64086-7)
Supplement: Supplementary file 2 — Description of Additional Supplementary Files [file 41467_2025_64086_MOESM2_ESM.pdf]

Description of Additional Supplementary Files

File Name: Supplementary Data 1.

Description: This separate archive (zip file) contains supplementary RAPDOR files used in the publication. All JSON files can be plugged into the RAPDOR Dash tool. Files are at least compatible with RAPDOR version 0.1.4. While they might work with other versions, we cannot guarantee that.

To learn about how to install RAPDOR and upload those files visit:

https://domonik.github.io/RAPDOR/

Files

synechoRAPDORGradRFile.json

The file for the *Synechocystis* 6803 GradR data. This is the same file that is displayed in our publication’s webserver.

HeLaEGFTreatment_egf_{x mins}.json

Pre-analyzed data for the reanalysis of data from Martinez-Val et al. (2021)[1]. Each file compares the protein distribution of HeLa cells x mins after EGF treatment to untreated cells.

Supplementary Data 2

Sheet 1. List of *Synechocystis* 6803 proteins detected by mass spectrometry. This data extends the data in **Fig. 1F**.

Sheet 2. Classification as candidate RBP or not in *Synechocystis* 6803 according to the TriPepSVM support vector machine classification. This data relates to **Supplementary Figure 6**.

Sheet 3. Overview on *Synechocystis* 6803 proteins detected in the GradR analysis. The table provides RAPDOR and locus IDs, gene names and protein functions in the first four columns, followed by the calculated mean distances, ANOSIM R values and ranks in the RAPDOR analysis (columns E to G). Whether a protein was predicted as an RBP by the SVM prediction and with which score and rank is indicated in columns H to J. The following columns indicate the R-DeeP significance as an RBP, followed by three columns with positions in the control and RNase-treated gradients and the relative fraction shifts. In columns O and P we indicate if a protein belongs to the ribosome and whether the respective MS data missed replicate(s). In columns Q and R we inform is the protein was selected for validation *in vivo* in a PNK assay (**Fig. 7**) and with what result. In the last column, we refer to a study in *Synechocystis* 6803 on the RNase P-interacting protein YlxR/Ssr1238[1], and to the results for putative homologs in a GradR analysis with the multicellular, N_2-fixing cyanobacterium *Nostoc* sp. PCC 7120[2]. Boldface letters indicate *Nostoc* homologs which were also shifting gradient fractions in the GradR experiment.

References in this file

1. Hemm, L. *et al.* Interactors and effects of overexpressing YlxR/RnpM, a conserved RNA binding protein in cyanobacteria. *RNA Biol.* **21**, 1308–1326 (2024).
2. Brenes-Álvarez, M. *et al.* R-DeeP/TripepSVM identifies the RNA-binding OB-fold-like protein PatR as regulator of heterocyst patterning. *Nucleic Acids Res.* **53**, gkae1247 (2025).
