# Supplementary material for: RAPDOR: Using Jensen-Shannon Distance for the computational analysis of complex proteomics datasets
Source: Nat Commun. 2025 Sep 26;16:8527. doi: 10.1038/s41467-025-64086-7 (PMC12475003; doi:10.1038/s41467-025-64086-7)
Supplement: Supplementary file 5 — Reporting Summary [file 41467_2025_64086_MOESM5_ESM.pdf]

nature portfolio

Corresponding author(s): Wolfgang R. Hess, Rolf Backofen

Last updated by author(s): Aug 27, 2025

Reporting Summary

Nature Portfolio wishes to improve the reproducibility of the work that we publish. This form provides structure for consistency and transparency in reporting. For further information on Nature Portfolio policies, see our Editorial Policies and the Editorial Policy Checklist.

Statistics

For all statistical analyses, confirm that the following items are present in the figure legend, table legend, main text, or Methods section.

n/a	Confirmed	
☐	☒	The exact sample size (n) for each experimental group/condition, given as a discrete number and unit of measurement
☐	☒	A statement on whether measurements were taken from distinct samples or whether the same sample was measured repeatedly
☐	☒	The statistical test(s) used AND whether they are one- or two-sided<br>*Only common tests should be described solely by name; describe more complex techniques in the Methods section.*
☐	☒	A description of all covariates tested
☐	☒	A description of any assumptions or corrections, such as tests of normality and adjustment for multiple comparisons
☐	☒	A full description of the statistical parameters including central tendency (e.g. means) or other basic estimates (e.g. regression coefficient) AND variation (e.g. standard deviation) or associated estimates of uncertainty (e.g. confidence intervals)
☐	☒	For null hypothesis testing, the test statistic (e.g. F, t, r) with confidence intervals, effect sizes, degrees of freedom and P value noted<br>*Give P values as exact values whenever suitable.*
☒	☐	For Bayesian analysis, information on the choice of priors and Markov chain Monte Carlo settings
☒	☐	For hierarchical and complex designs, identification of the appropriate level for tests and full reporting of outcomes
☐	☒	Estimates of effect sizes (e.g. Cohen's d, Pearson's r), indicating how they were calculated

Our web collection on statistics for biologists contains articles on many of the points above.

Software and code

Policy information about availability of computer code

Data collection	Mass spectrometry data was acquired using an LTQ Orbitrap Velos Pro (Thermo Fisher Scientific, USA).<br>Northern blot hybridization was visualized with the Typhoon FLA 9500 (GE Healthcare Life Sciences) scanner and the Typhoon FLA 9500 software (version 1.1).<br>Western blot signals were detected with Fusion SL4 and the according Fusion Capt Advanced SL7 software (version17.04a).<br>Pre analyzed data for the spatial proteomics were taken from:<br>https://doi.org/10.1038/s41467-021-27398-y<br>and<br>https://doi.org/10.1021/acs.jproteome.2c00759<br>GO Terms for Synechocystis sp. PCC 6803 were downloaded from UniProt at https://www.uniprot.org/.
Data analysis	Proteomics data was analyzed using Perseus (v2.0.3.0) and MaxQuant (version 2.0.3.0).<br>All further GradR analysis was documented via a snakemake workflow available via:<br>https://github.com/domonik/synRDPMSpec and DOI: 10.5281/zenodo.16947727 and DOI:10.5281/zenodo.16947957<br>This also contains MaxQuant pre analyzed data for reproducibility.<br>Tool versions used are documented in the GitHub repository in the main branch at ./envs/*.pin.txt<br>or inside the environment.pin.txt file.<br>The regression analysis was conducted using custom R code.<br>The generated proteomics data can be accessed at https://www.ebi.ac.uk/pride/archive/projects/PXD045848.<br>The analyzed Synechocystis 6803 data can be accessed under: https://synecho-rapdor.biologie.uni-freiburg.de

Northern blot and western blot signal intensities were analyzed with Quantity One (version 4.6.6).
All statistics were calculated with Excel, data and plots were visualized with OriginPro (2022) software (version 9.9.0.225) and vector images were created with Inkscape (version 1.2.1).
The TripepSVM algorithm was developed and published previously (Bressin, A. et al. Nucleic Acids Res. 47, 4406–4417 (2019)). It is available at github (https://github.com/marsicoLab/TriPepSVM).
The RAPDOR tool is available as a pypi package and its documentation is hosted onGitHub at https://github.com/domonik/RAPDOR
The code used to analyze the data including the modified R-DeeP script is available as a snakemake workflow on Github: (https://github.com/domonik/synRDPMSpec).

For manuscripts utilizing custom algorithms or software that are central to the research but not yet described in published literature, software must be made available to editors and reviewers. We strongly encourage code deposition in a community repository (e.g. GitHub). See the Nature Portfolio guidelines for submitting code & software for further information.

Data

Policy information about availability of data

All manuscripts must include a data availability statement. This statement should provide the following information, where applicable:

- Accession codes, unique identifiers, or web links for publicly available datasets
- A description of any restrictions on data availability
- For clinical datasets or third party data, please ensure that the statement adheres to our policy

The datasets produced in this study are available in the following databases:
● Mass spectrometry raw data were deposited at the ProteomeXchange (15) Consortium (http://proteomecentral.proteomexchange.org) via the PRIDE partner repository (16) under the identifier PXD045848 [https://www.ebi.ac.uk/pride/archive/projects/PXD045848].
● Synechocystis 6803 data accessibility and visualization: https://synecho-rapdor.biologie.uni-freiburg.de
● The RAPDOR tool is available as a pypi package and its documentation is hosted on https://domonik.github.io/RAPDOR/
● The code used to analyze the data including the modified R-DeeP script is available as a snakemake workflow on Github: (https://github.com/domonik/synRDPMSpec) and DOI: 10.5281/zenodo.16947727

Research involving human participants, their data, or biological material

Policy information about studies with human participants or human data. See also policy information about sex, gender (identity/presentation), and sexual orientation and race, ethnicity and racism.

Reporting on sex and gender	No research involving human research participants.
Reporting on race, ethnicity, or other socially relevant groupings	N/A
Population characteristics	N/A
Recruitment	N/A
Ethics oversight	N/A

Note that full information on the approval of the study protocol must also be provided in the manuscript.

Field-specific reporting

Please select the one below that is the best fit for your research. If you are not sure, read the appropriate sections before making your selection.

[x] Life sciences [ ] Behavioural & social sciences [ ] Ecological, evolutionary & environmental sciences

For a reference copy of the document with all sections, see nature.com/documents/nr-reporting-summary-flat.pdf

Life sciences study design

All studies must disclose on these points even when the disclosure is negative.

Sample size	The current work doesn't involve the analysis of populations. For functional assays, the number of times that experiments were repeated is addressed under "Replication" below.
Data exclusions	No data was excluded.
Replication	Experiments were conducted using replicates. The detailed replicates for each experiments are given in the manuscript. All attempts at replication were successful.
Randomization	Assigning cell cultures to RNase treated vs. control group was done at random. Later all replicates were processed in parallel in order to reduce technical variability.

Blinding	Investigators were not blinded to group allocation because group allocation was not involved in our study. Investigators were not blinded during data collection because the collected data were quantitative in nature (such as bands in gel blot analyses or intensity values) and were not prone to subjective interpretation.

Reporting for specific materials, systems and methods

We require information from authors about some types of materials, experimental systems and methods used in many studies. Here, indicate whether each material, system or method listed is relevant to your study. If you are not sure if a list item applies to your research, read the appropriate section before selecting a response.

Materials & experimental systems

n/a	Involved in the study	
☐	☒	Antibodies
☒	☐	Eukaryotic cell lines
☒	☐	Palaeontology and archaeology
☐	☒	Animals and other organisms
☒	☐	Clinical data
☒	☐	Dual use research of concern
☒	☐	Plants

Methods

n/a	Involved in the study	
☒	☐	ChIP-seq
☒	☐	Flow cytometry
☒	☐	MRI-based neuroimaging

Antibodies

Antibodies used	Agrisera Anti-RPL1 | 50S ribosomal protein L1 Product No. AS11 1738; Mouse monoclonal anti-FLAG antiserum conjugated to horseradish peroxidase (ANTI-FLAG® M2-Peroxidase,# A8592 Sigma-Aldrich).
Validation	The antisera were tested for Westernblots by the manufacturers.

Animals and other research organisms

Policy information about studies involving animals; ARRIVE guidelines recommended for reporting animal research, and Sex and Gender in Research

Laboratory animals	Synechocystis sp. PCC 6803 (a cyanobacterium)
Wild animals	N/A
Reporting on sex	N/A
Field-collected samples	N/A
Ethics oversight	N/A

Note that full information on the approval of the study protocol must also be provided in the manuscript.

Plants

Seed stocks	This study did not involve research on plants.
Novel plant genotypes	N/A
Authentication	N/A
